# Supplementary material for: Multi‐dataset identification of innovative feature genes and molecular mechanisms in keratoconus
Source: J Cell Mol Med. 2024 Sep 19;28(18):e70079. doi: 10.1111/jcmm.70079 (PMC11412914; doi:10.1111/jcmm.70079)
Supplement: Supplementary file 2 — Material S2. [file JCMM-28-e70079-s002.zip › 7.GSEA analysis/5.RNF217-AS1/KEGG_CYSTEINE_AND_METHIONINE_METABOLISM.html]

Details for gene set KEGG\_CYSTEINE\_AND\_METHIONINE\_METABOLISM[GSEA]

|  || Dataset | RNF217-AS1.group.cls#h\_versus\_l |
| Phenotype | group.cls#h\_versus\_l |
| Upregulated in class | h |
| GeneSet | KEGG\_CYSTEINE\_AND\_METHIONINE\_METABOLISM |
| Enrichment Score (ES) | 0.21874693 |
| Normalized Enrichment Score (NES) | 0.9685245 |
| Nominal p-value | 0.49423078 |
| FDR q-value | 1.0 |
| FWER p-Value | 0.999 |
Table: GSEA Results Summary

  

Fig 1: Enrichment plot: KEGG\_CYSTEINE\_AND\_METHIONINE\_METABOLISM      
 Profile of the Running ES Score & Positions of GeneSet Members on the Rank Ordered List

  

| SYMBOL | TITLE | RANK IN GENE LIST | RANK METRIC SCORE | RUNNING ES | CORE ENRICHMENT || 1 | IL4I1 | na | 570 | 0.516 | 0.1308 | Yes |
| 2 | TAT | na | 1456 | 0.397 | 0.2187 | Yes |
| 3 | BHMT | na | 5787 | 0.190 | 0.1482 | No |
| 4 | LDHAL6A | na | 6613 | 0.164 | 0.1714 | No |
| 5 | CDO1 | na | 7373 | 0.143 | 0.1903 | No |
| 6 | DNMT3A | na | 9857 | 0.089 | 0.1440 | No |
| 7 | SMS | na | 11913 | 0.057 | 0.1009 | No |
| 8 | DNMT3L | na | 12859 | 0.044 | 0.0863 | No |
| 9 | MPST | na | 13368 | 0.038 | 0.0824 | No |
| 10 | AHCY | na | 13659 | 0.034 | 0.0839 | No |
| 11 | MAT2A | na | 13771 | 0.033 | 0.0901 | No |
| 12 | MTAP | na | 14832 | 0.021 | 0.0655 | No |
| 13 | LDHA | na | 16042 | 0.008 | 0.0330 | No |
| 14 | LDHB | na | 16493 | 0.004 | 0.0211 | No |
| 15 | GOT2 | na | 17814 | -0.009 | -0.0143 | No |
| 16 | AHCYL1 | na | 18071 | -0.011 | -0.0184 | No |
| 17 | MAT2B | na | 18082 | -0.012 | -0.0154 | No |
| 18 | MAT1A | na | 18213 | -0.013 | -0.0155 | No |
| 19 | GOT1 | na | 20120 | -0.031 | -0.0616 | No |
| 20 | AMD1 | na | 20688 | -0.037 | -0.0674 | No |
| 21 | ENOPH1 | na | 22946 | -0.061 | -0.1150 | No |
| 22 | CBS | na | 23765 | -0.072 | -0.1181 | No |
| 23 | TRDMT1 | na | 24239 | -0.079 | -0.1092 | No |
| 24 | APIP | na | 24593 | -0.084 | -0.0954 | No |
| 25 | CTH | na | 25070 | -0.091 | -0.0831 | No |
| 26 | LDHC | na | 25553 | -0.099 | -0.0686 | No |
| 27 | ADI1 | na | 26106 | -0.109 | -0.0534 | No |
| 28 | LDHAL6B | na | 26559 | -0.117 | -0.0329 | No |
| 29 | MTR | na | 27079 | -0.127 | -0.0115 | No |
| 30 | DNMT1 | na | 27702 | -0.141 | 0.0109 | No |
| 31 | SRM | na | 28051 | -0.150 | 0.0437 | No |
| 32 | AHCYL2 | na | 28262 | -0.155 | 0.0819 | No |
| 33 | DNMT3B | na | 28950 | -0.175 | 0.1120 | No |
| 34 | SDS | na | 29501 | -0.191 | 0.1507 | No |
Table: GSEA details [plain text format]

  

Fig 2: KEGG\_CYSTEINE\_AND\_METHIONINE\_METABOLISM      
 Blue-Pink O' Gram in the Space of the Analyzed GeneSet

  

Fig 3: KEGG\_CYSTEINE\_AND\_METHIONINE\_METABOLISM: Random ES distribution      
 Gene set null distribution of ES for **KEGG\_CYSTEINE\_AND\_METHIONINE\_METABOLISM**
